# Uncovering hidden immune defects in childhood granulomatous disorders: a case report

**DOI:** 10.3389/fimmu.2025.1634661

**Published:** 2025-07-18

**Authors:** Walter Maria Sarli, Francesca Quaranta, Clementina Canessa, Lorenzo Lodi, Laura Pisano, Anna Maria Buccoliero, Teresa Oranges, Elena Sieni, Gabriele Simonini, Luca Bartolini, Elisabetta Venturini, Luisa Galli, Chiara Azzari, Silvia Ricci

**Affiliations:** ^1^ Department of Health Sciences, University of Florence, Florence, Italy; ^2^ Immunology Unit, Meyer Children’s Hospital IRCCS, Florence, Italy; ^3^ Pathology Unit, Meyer Children’s Hospital IRCCS, Florence, Italy; ^4^ Dermatology Unit, Meyer Children’s Hospital, IRCCS, Florence, Italy; ^5^ Pediatric Hematology-Oncology Department, Meyer Children’s Hospital IRCCS, Florence, Italy; ^6^ Rheumatology Unit, Meyer Children’s Hospital IRCCS, Florence, Italy; ^7^ Department Neurosciences, Psychology, Drug Research and Child Health (NEUROFARBA), University of Florence, Florence, Italy; ^8^ Neuroscience Department, Meyer Children’s Hospital IRCCS, Florence, Italy; ^9^ Infectious Disease Unit, Meyer Children’s Hospital IRCCS, Florence, Italy

**Keywords:** Mendelian susceptibility to mycobacterial diseases, innate immunity, STAT1, sarcoidosis, mycobacterium avium complex Mendelian susceptibility to mycobacterial diseases (MSMD), mycobacterium avium complex (MAC), nontuberculous mycobacteria (NTM), interferon-gamma (IFNg)

## Abstract

Granulomatous diseases in childhood present a complex diagnostic landscape, particularly when histological and clinical findings overlap with those of systemic inflammatory or histiocytic disorders. A subset of these conditions may represent the clinical onset of inborn errors of immunity (IEI), such as Mendelian Susceptibility to Mycobacterial Disease (MSMD), where atypical or sterile granulomas may obscure the underlying infectious or genetic etiology. Recognition of IEI behind granulomatous diseases can radically alter patient’s prognosis and therapeutic management. This report describes the case of a 11-years-old with an initial diagnosis of Rosai-Dorfman disease based on clinical and and histological findings. Following relapse after steroid tapering the diagnosis was revised to sarcoidosis, supported by non-caseating granulomas and compatible laboratory findings. Only after cultures from biopsy specimens revealed *Mycobacterium avium* complex (MAC), immunological investigations were undertaken, revealing a STAT1 dominant negative deficiency, consistent with MSMD. This report underscores the need of considering IEI in pediatric patients presenting with granulomatous inflammation, especially when clinical course is atypical or refractory to standard immunosuppressive therapies. Early microbiological and immunogenetic assessment is essential to avoid diagnostic delay, prevent inappropriate treatment, and guide targeted antimicrobial therapy.

## Introduction

Granulomatous diseases encompass a wide range of conditions defined by the formation of inflammatory infiltrates of macrophages, epithelioid cells, and multinucleated giant cells, surrounded by fibroblasts and lymphocytes. The differential diagnosis of granulomatous diseases in children is particularly challenging, as granulomas can form in response to a broad spectrum of triggers, such as infectious agents, as well as non-infectious causes, including malignancies, inborn errors of immunity (IEI), rare histiocytic disorders such as Rosai-Dorfman disease – characterized by infiltration of lymph nodes or extra-nodal tissues by non-malignant histiocytes – and systemic inflammatory disorders (1, 2). Among these, sarcoidosis poses a particular diagnostic challenge, due to its nonspecific clinical presentations, lack of pathognomonic signs, and the absence of reliable laboratory tests (3–5). Sarcoidosis is a chronic multisystem inflammatory disease which can potentially affect various organs, including the skin, lungs, kidneys, bones, reticuloendothelial and central nervous system (6, 7). While it is more commonly seen in adults, pediatric cases are rare, with an estimated incidence of 0.22-0.29 per 100,000 children (8, 9). The diagnosis of sarcoidosis is further complicated by the fact that sarcoid-like lesions can also result from infections, such as mycobacteria, which are more commonly encountered in pediatric patients. This is particularly difficult in those with underlying IEI, such as Mendelian Susceptibilities to Mycobacterial Diseases (MSMD), where typical histological features of mycobacterial infection may be absent.

MSMD are IEI affecting the interaction between mononuclear phagocytes and T-helper cells just as their response to interleukin-12/interferon-gamma (IL12/IFNγ) (10). This leads to susceptibility to intracellular pathogens like mycobacteria, but also infections by fungi, *Salmonella* spp, *Burkholderia* spp, *Listeria* spp, and herpes viruses. Since the first report of familial disseminated atypical mycobacteriosis in 1964 (11), interest in MSMD has gained momentum, driven by advances in genetic research and laboratory diagnostics. As of 2022, 17 single-gene defects have been identified within the MSMD subgroup (12), though genetic causes remain unknown in nearly 50% of cases (13).

Among the known MSMD genes, signal transducer and activator of transcription 1 (*STAT1*) encodes a protein composed of four domains, that upon IFNγ stimulation, undergoes phosphorylation at Tyr701, homodimerization and translocation to the nucleus, playing a critical role in the defense against various pathogens, primarily mycobacteria (14).

*STAT1* deficiencies can be autosomal recessive (AR) or autosomal dominant (AD). AR *STAT1* deficiencies may be hypomorphic (partial), leading to milder MSMD forms, or amorphic (complete), which often results in death during infancy due to disseminated Bacillus Calmette-Guerin (BCG) or viral infections unless treated with hematopoietic stem cell transplantation (HSCT) (15). Conversely, AD *STAT1* deficiencies generally cause less severe infections in childhood, as they exert a milder effect on the IFNα/β signaling pathway, with BCGitis, multifocal osteomyelitis and *Mycobacterium avium complex* (MAC) infections representing the most common clinical manifestations, as demonstrated by a recent comprehensive review, which analyzed 24 cases of AD *STAT1* deficiencies (16).

We report a case of a previously healthy female who, due to the nonspecific clinical and histological features, was initially misdiagnosed with histiocytosis, later with sarcoidosis, and ultimately found to harbor AD *STAT1* deficiency following the pivotal microbiological identification of MAC from one of her lesions.

## Case presentation

**Figure 1:**
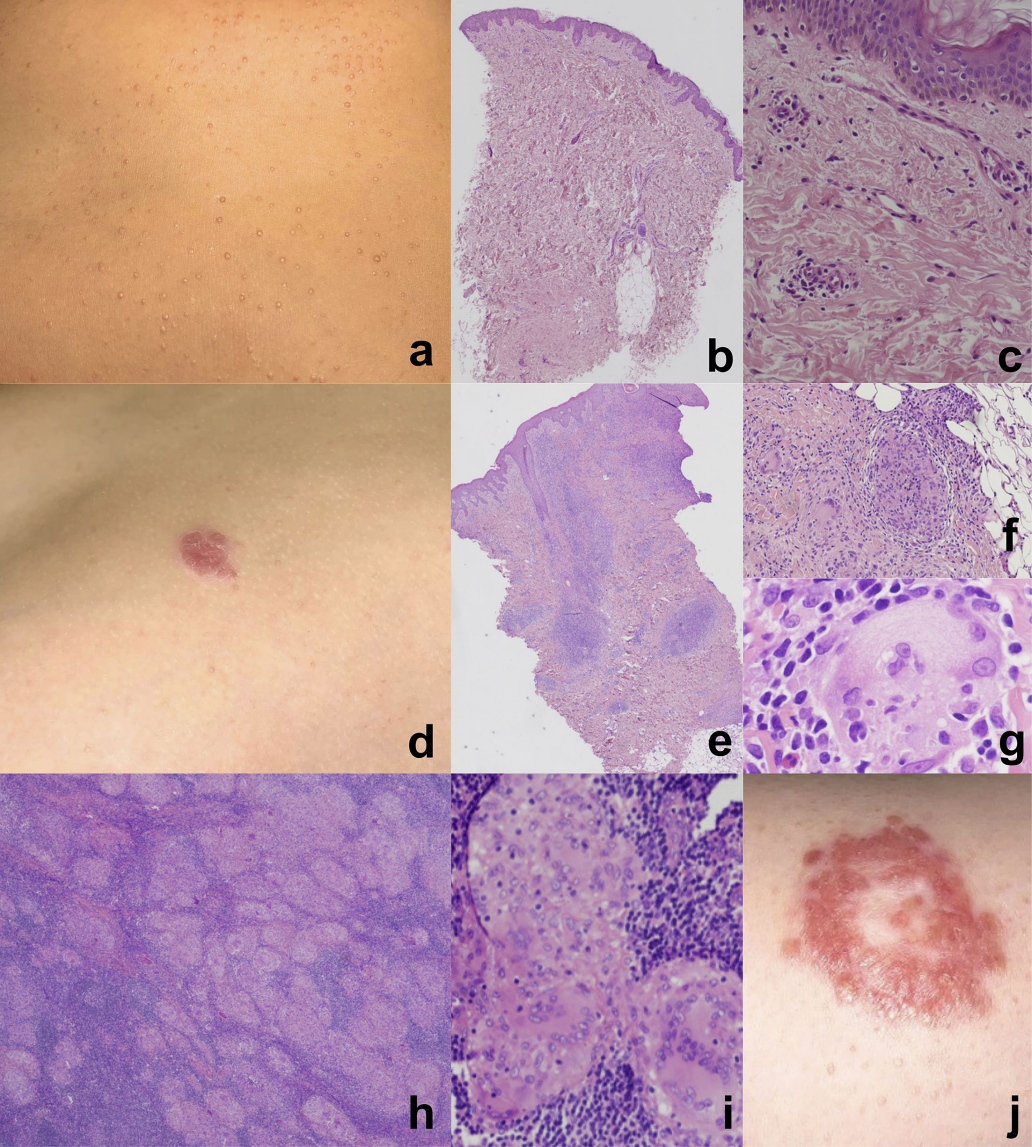
Multiple follicular papules on the trunk **(a)** in a 9 years-old female affected by *Mycobacterium avium complex* (MAC) infection. Subtle and nonspecific histopathological alterations. Hematoxylin and eosin staining, original magnification: **(b)** 4x, **(c)** 20x. Erythematous plaque on the abdominal region of the same patient at the age of 11 years **(d)**. Granulomatous inflammatory infiltrate with histiocytic-macrophagic cells **(e, f)** exhibiting emperipolesis **(g)**, extending throughout the entire dermis **(e)**. Hematoxylin and eosin staining, original magnification: **(e)** 4x, **(f)** 10x, **(g)** 60x. Multiple non-necrotizing epithelioid granulomas. Hematoxylin and eosin staining, original magnification: **(h)** 2x, **(i)** 10x. Large erythematous plaque on the trunk with annular shape, four months after antibacterial treatment and during steroid tapering **(j)**. The lesion shows peripheral scaling and central clearing, giving a targetoid appearance.

The patient is a female and first child of a healthy, non-consanguineous Italian family. Her past clinical history was notable for two episodes of viral pneumonia, without complications, and for the onset of multiple follicular papules at the age of 7 years, which first involved the knees and then spread to the trunk and face, gradually worsening over time (**Figure 1a**). Histopathological analysis of an initial skin biopsy demonstrated very subtle and nonspecific alterations, characterized by minimal spongiosis and few mixed perivascular inflammatory infiltrates (**Figures 1b, c**). However, a second biopsy at the age of 11 years, following the emergence of erythematous plaques on the trunk (**Figure 1d**), revealed a dense BRAF600E-negative lymphoplasmacytic inflammatory infiltrate with histiocytic-macrophagic S100-positive cells and emperipolesis (**Figures 1e-g**). These histological findings raised the possibility of Rosai-Dorfman disease.

**Figure 2:**
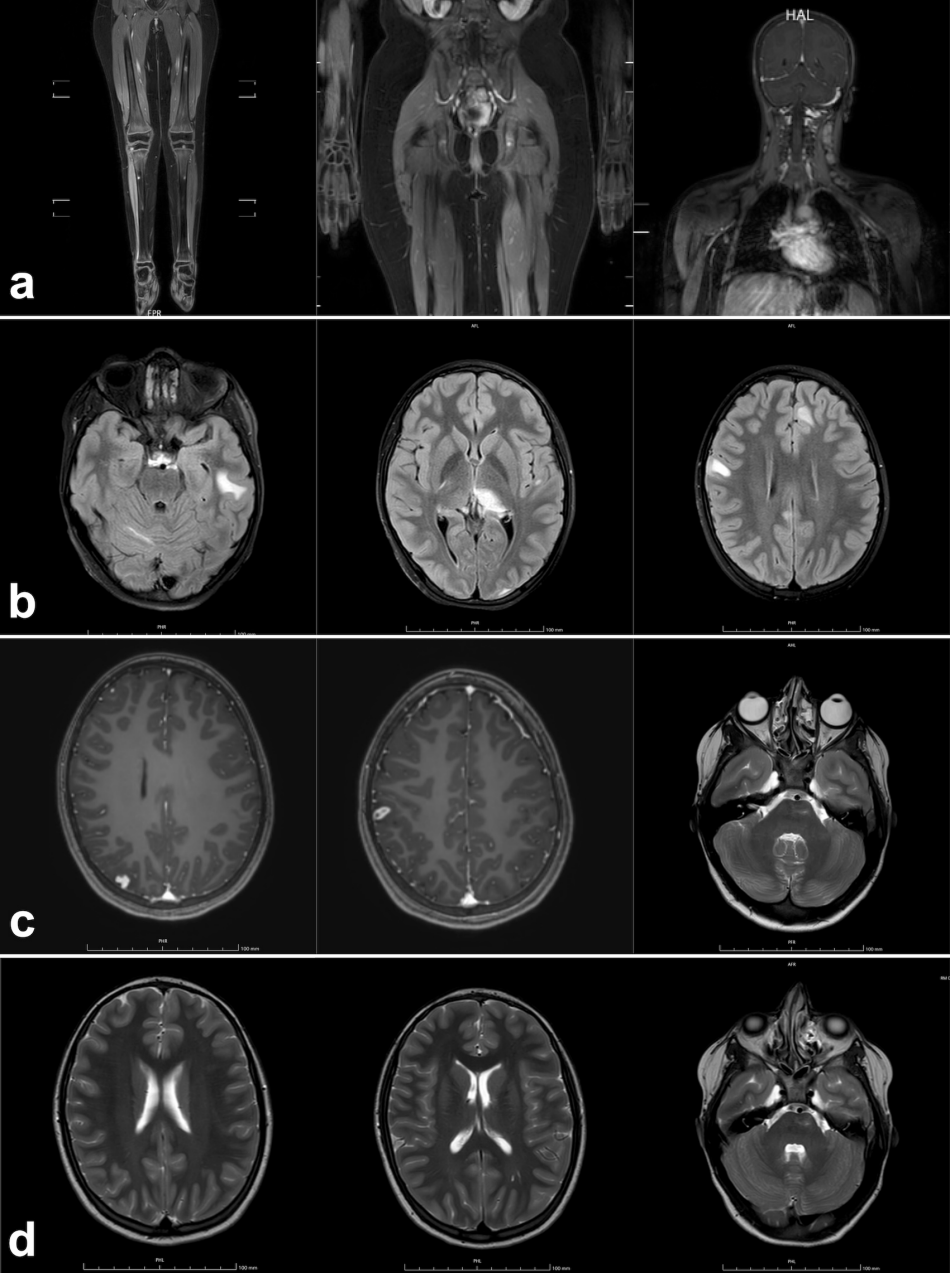
Detail of the initial whole-body MRI of a 11 years-old female patient with Mycobacterium avium complex (MAC) infection and sarcoid-like lesions, showing multiple bone lesions in the tibial diaphyses and epiphyses, left femoral diaphysis, ischial punctate lesions, and left cervical lymph node involvement **(a)**. Brain MRI performed during disease exacerbation, 9 months after the initial whole-body MRI, revealed multiple areas of signal alteration in the right cerebellar hemisphere, left temporal and frontal subcortical white matter, splenium, and left thalamus **(b)**. Overall reduction of the previously noted areas of signal alteration, apart from a small new hyperintense lesion in the right parietal region **(c)**, observed 4 months after the initiation of triple antimycobacterial therapy. Further reduction of the known alterations was noted, with stable size of the left pontine lesion **(d)**, assessed 3 months later.

A whole-body magnetic resonance imaging (WB-MRI) for staging (**Figure 2a**) showed multiple hyperintense lesions in long bones and skull, and lymph node enlargements, particularly in cervical region. Thus, the patient was started on oral steroids (prednisone 1 mg/kg/day), then tapered over six weeks, with clinical improvement. Follow-up WB-MRIs showed progressive partial regression of bone lesions and an overall reduction of lymph node size. After discontinuation of treatment, the patient remained asymptomatic for nine months, at which point disease relapsed with new skin flare, multiple lymph node and bone lesions, and brain involvement confirmed on MRI spectroscopy (**Figure 2b**), although the patient remained neurologically asymptomatic. Oligoclonal bands, Link index, and autoantibodies (anti-myelin oligodendrocyte glycoprotein, and anti-aquaporin4) on cerebrospinal fluid were found to be normal.

The partial response to steroid therapy, combined with the atypical clinical presentation, prompted a reconsideration of the initial presumptive diagnosis of Rosai-Dorfman disease. Thus, for histological redefinition and molecular deepening, the patient underwent a lymph node biopsy, revealing multiple non-necrotizing epithelioid granulomas, with multinucleated S100+ macrophages and emperipolesis. Although the findings were not definitively diagnostic, they were highly suggestive of sarcoidosis (**Figures 1h, i**). This diagnosis was then confirmed by Expert review at the European reference center for rare histiocytoses. Laboratory results supported the latter histological findings. showed nonspecific pulmonary lesions.

Despite the absence of significant pulmonary involvement on high resolution computed tomography or lung functionality, the patient was treated as for sarcoidosis with a new course of oral steroids (prednisone 1 mg/kg/day), leading to marked improvement in skin lesions and cervical lymph node size.

As the initial findings did not suggest an infectious etiology, microbiological cultures were not initially performed. However, given disease progression, these were later conducted on the biopsy specimen, revealing *Mycobacterium avium* complex (MAC). Thus, the patient started triple oral antimycobacterial therapy including clarithromycin (15 mg/kg/day), ethambutol (15 mg/kg/day), and moxifloxacin (8 mg/kg/day) then replaced with rifampicin (15 mg/kg/day) after 4 weeks to minimize potential side effects of fluoroquinolones. No side effects were reported.

In the suspicion of an underlying IEI, an extensive immunological work-up was performed (**Supplementary Table S1**), which revealed absent STAT1 phosphorylation after IFNγ stimulation (**Supplementary Figure A**), confirmed during steroid tapering.

Targeted next-generation sequencing identified two *STAT1* variants. The first (c.1823G>A, p.R608Q) was a known pathogenic dominant-negative loss-of-function (LOF) variant previously reported in MSMD (17). The second (c.129-9A>G) was a variant of unknown significance (VUS), initially considered potentially pathogenic due to its rarity and location at a splicing site, but later reconsidered as this one was also found in the healthy mother. Clinical exome sequencing excluded pathogenic variants or VUS in genes linked to histiocytic disorders and granulomatous autoinflammatory diseases, including Blau syndrome.

Four months after starting antibiotics and during steroid tapering, mild skin lesions persisted without evident improvement (**Figure 1j**); however, WB-MRI showed overall improvement (**Figure 2c**), and no systemic signs of disease activity were observed. Consequently, alternative therapeutic options, such as IFNγ, were considered but not administered, given the favorable radiological response. Moxifloxacin was reintroduced to enhance CNS penetration and strengthen MAC treatment, while steroid therapy was discontinued. The patient remained asymptomatic and did not experience any new relapse following steroid discontinuation. Follow-up brain MRIs, conducted every three months, showed progressive regression of all previously identified lesions (**Figure 2d**). Moxifloxacin was stopped after 9 months, and triple therapy after 13 months, followed by ongoing azithromycin prophylaxis. After 18 months, the patient remains in excellent clinical conditions with no infections reported. Azithromycin prophylaxis is planned to continue for an extended period to prevent relapse, with close clinical, laboratory and radiological monitoring. The long-term prognosis remains cautiously optimistic given the patient’s favorable response to therapy so far.

**Figure 3:**
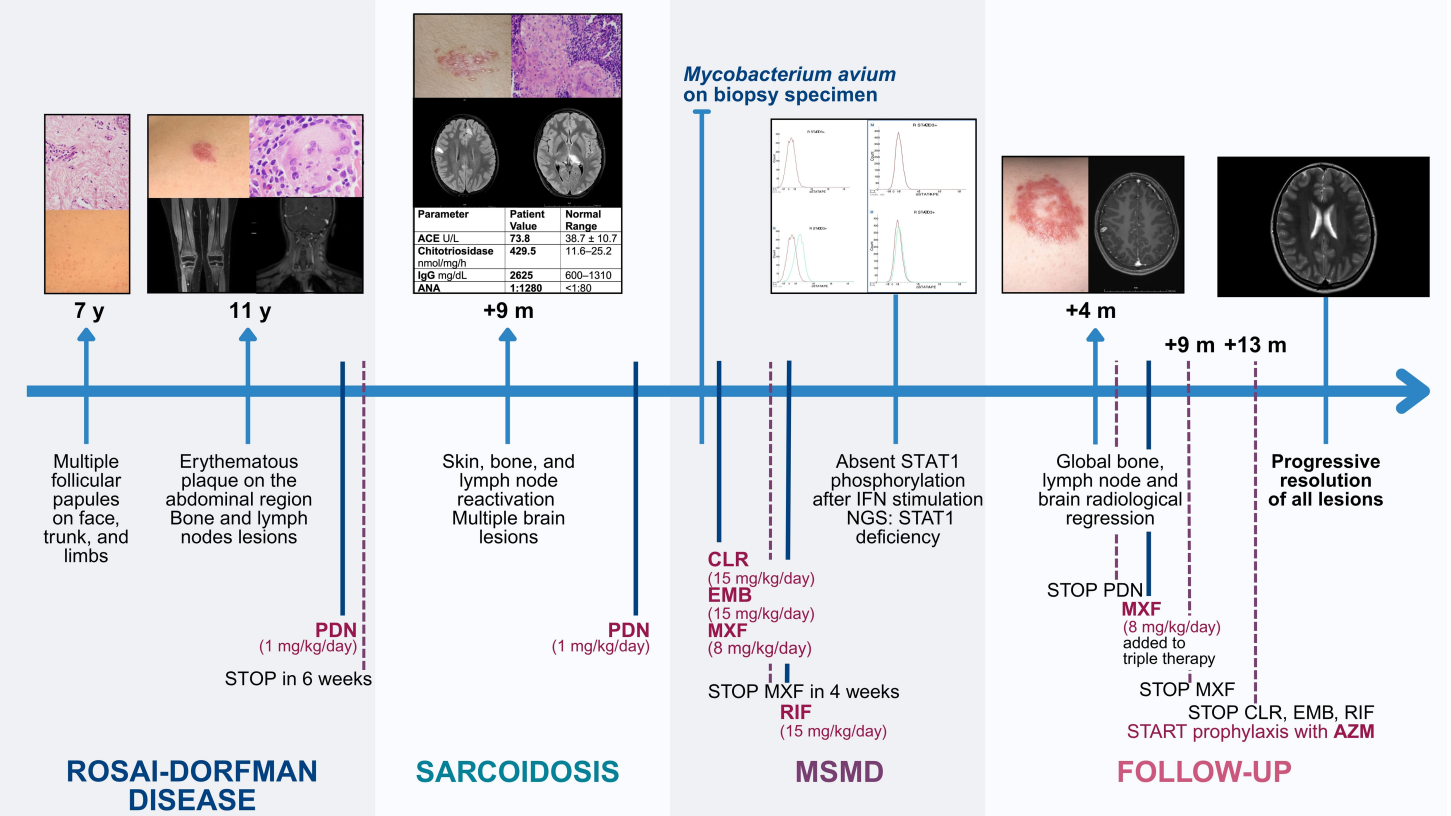
Clinical timeline of the patient’s medical history. A chronological overview of the patient’s clinical progression, starting from the onset of dermatological symptoms at age 7. ACE, angiotensin converting enzyme; ANA, anti-nuclear antibodies; AZM, azithromycin; CLR, clarithromycin; EMB, ethambutol; IFN, interferon; IgG, immunoglobulin G; m, months; MSMD, Mendelian Susceptibility to Mycobacterial Diseases; MXF, moxifloxacin; NGS, next generation sequencing; PDN, prednisone; RIF, rifampicin; STAT1, signal transducer and activator of transcription 1; y, years.

A chronological overview of the patient’s clinical progression is represented in **Figure 3**.

## Discussion

Pediatric granulomatous disorders pose a significant diagnostic challenge because of their rarity, lack of pathognomonic features, and overlapping clinical presentations. In these circumstances, pediatricians should consider IEI as a possible underlying cause, especially when granulomas are sterile or display atypical features.

Granulomas are reported in approximately 1–4% of IEI (18) and can be either infectious or sterile in origin. In disorders such as common variable immunodeficiency (CVID), combined immunodeficiency (CID), autoinflammatory disorders (19) and sometimes chronic granulomatous disease (CGD) (20), granulomas often result from sterile hyperinflammatory response. On the contrary, in conditions like MSMD, defective immune responses lead to chronic infections by tuberculous and non-tuberculous mycobacteria (19), fungi (21), or viruses (22).

One of the key diagnostic difficulties lies in the fact that granulomas observed in these immune defects, whether infectious or sterile, may closely mimic those seen in other granulomatous disorders. For example, sarcoid-like lesions have been reported in CID due to RAG1/2 mutations, whereas PRKCD deficiency has been associated with non-Langerhans histiocytosis (23). Syndromic CID, such as ataxia-telangiectasia or cartilage-hair hypoplasia, may also present with sarcoid-like lesions or histiocytic infiltrates (23).

This diagnostic overlap could result in microbial cultures of histological specimens not being systematically pursued in routine clinical practice.

A key strength of this report is the clear retrospective distinction between the final diagnosis of MSMD and other granulomatous conditions like Rosai-Dorfman disease or sarcoidosis.

Our patient initially presented with complex clinical and histological features suggestive of Rosai-Dorfman disease, supported by typical cervical lymph node and long bone involvement (24). Later, disease flare after steroid withdrawal and central nervous system involvement prompted diagnostic reassessment, with sarcoidosis considered in light of new supportive histological and laboratory findings. However, the presentation was atypical, characterized by prominent skeletal involvement (25) and the absence of clear pulmonary (26), articular, and ocular manifestations (27).

The patient’s therapeutic course provided further insight. Initial steroid therapy achieved transient disease control, as expected firstly in Rosai-Dorfman disease then in sarcoidosis. However, closer evaluation suggests this response may have reflected the steroids’ ability to modulate granulomatous inflammation, a known effect in mycobacterial infections (28, 29). Although we cannot definitively ascertain if MAC was present in all granulomas, the progressive resolution of the lesions with antimycobacterial therapy alone suggests that the disease was not purely inflammatory, as initially suspected. Whether it was disseminated mycobacterial disease or an inflammatory response triggered by MAC, in either case, the elimination of the infection led to clinical resolution. This observation is critical, as a pure sarcoidosis would not have improved, but even worsened, following steroid discontinuation.

This clinical course underscores a diagnostic consideration: granulomatous diseases that don’t show sustained improvement with immunosuppression should prompt deep investigation for underlying infections or immune deficiencies.

This is particularly relevant in IEI such as MSMD, which selectively affect innate immunity, where infectious history or laboratory assessments often lack suggestive hallmarks for immunodeficiency. Notably, delayed complications following BCG vaccination—often among the earliest clinical features of MSMD—may go undocumented, as in our patient, due to the absence of routine BCG immunization in countries like Italy.

These diagnostic ambiguities raise broader questions about the interplay between granulomatous inflammation, infectious triggers, and immunity, particularly in conditions like sarcoidosis, whose etiology remains poorly understood. IFNγ signaling, essential for the immune response to mycobacteria, also appears to play a key pathogenic role across multiple granulomatous disorders. This is supported by a recent case describing a patient with a milder form of Blau syndrome – a monogenic granulomatous disease - due to the interference of a coexisting dominant-negative *IFNGR1* mutation (30).

The relationship between sarcoidosis, mycobacterial infections, and immune dysregulation has been debated for decades (31, 32). This association dates back to 1960, when *Mycobacterium tuberculosis* was first reported in multiple patients with sarcoidosis (33). More recently, NTM have been detected in patients meeting clinical and histopathological criteria for sarcoidosis in many reports in the literature (34–38). Notably, when the histology was described, necrosis was not present in the granulomas. In most cases however, genetic investigations to rule out an underlying IEI were not conducted.

**Figure 4:**
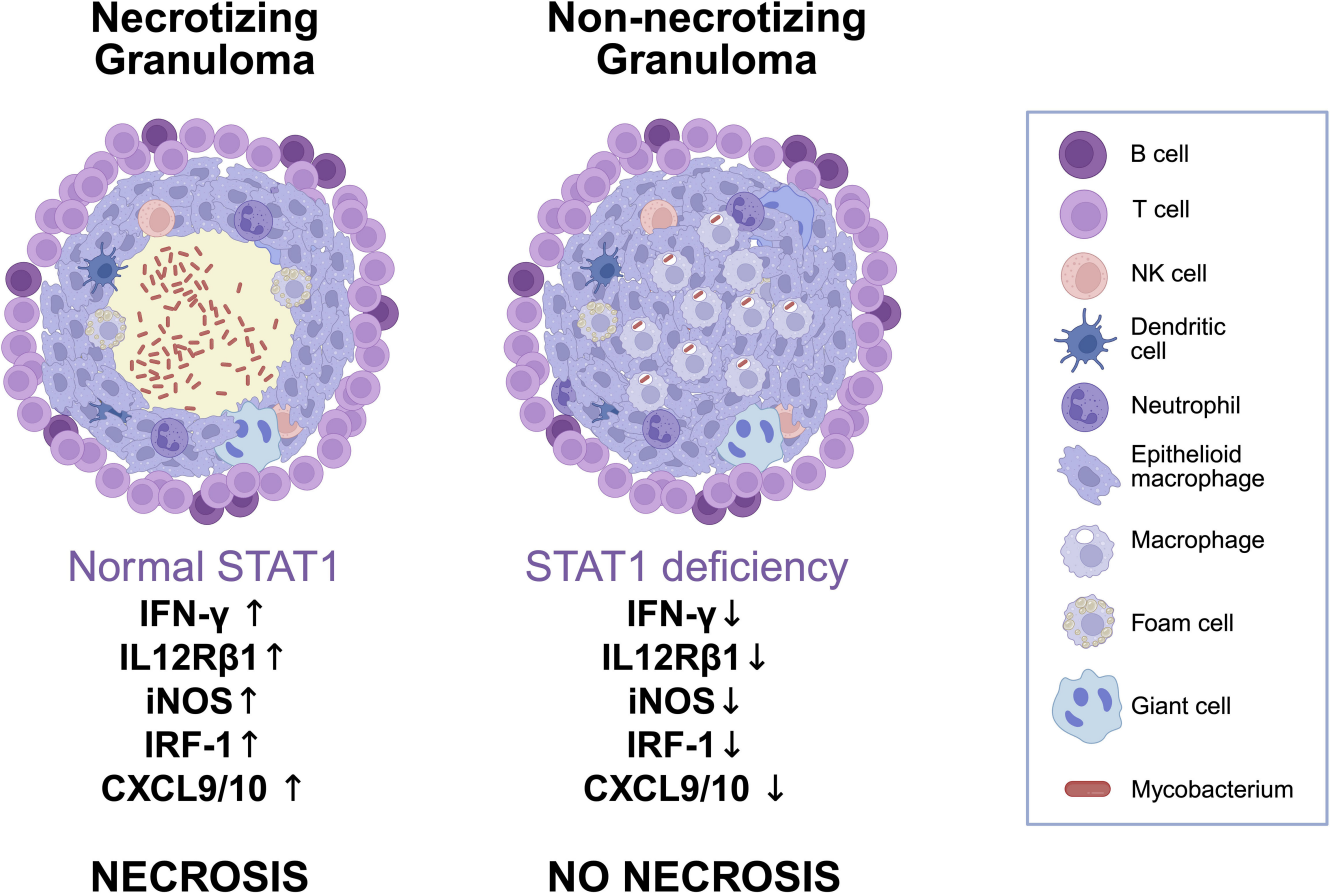
Schematic comparison of granuloma architecture and immune signaling in normal versus STAT1-deficient conditions. Comparison of granulomas in normal versus STAT1-deficient immunity. Functional STAT1 supports IFNγ signaling, leading to strong Th1 responses, macrophage activation, and caseating necrosis. In STAT1 deficiency, impaired signaling reduces iNOS, IRF1, CXCL9/10, and IL12Rβ1 expression, resulting in non-necrotizing granulomas with poor immune containment of mycobacteria and possible overalapping histological features with other granulomatous diseases such as sarcoidosis. Created in BioRender. Ricci, S. (2025) https://BioRender.com/urn1gx7.

NTM infections are often associated with non-necrotizing granulomas, particularly in individuals immunocompromised by Human Immunodeficiency Virus (HIV) or immunosuppressive drugs, where histological findings may be nonspecific (39). In our case, MAC was isolated from a non-caseating granuloma (**Figure 4**), initially interpreted as consistent with sarcoidosis, prompting further immunological assessment that ultimately led to the diagnosis of MSMD. Thus, it could be speculated that some sarcoidosis diagnoses, based on clinical, laboratory and histological criteria may hide unidentified mycobacterial infections or genetically defined IEI. In 2022, in effect, sarcoid-like lesions were described in a 31-year-old woman with disseminated *Mycobacterium genavense* infection then found to be due to IL12Rβ1 deficiency (40). Retrospectively, and in light of our case, it cannot be ruled out that other cases previously described in the literature may have hidden unrecognized MSMD.

The absence of necrotic areas in granulomas has been supported by STAT1-knockout mouse models (41). Similarly, cases of patients with dominant negative STAT1 LOF mutations also document non-necrotic granulomas in the context of mycobacterial infections (16, 42). However, this is not universal, as residual STAT1 function in some cases may allow necrosis to form, emphasizing the heterogeneity of clinical presentations (43, 44).

In pediatric patients with sarcoidosis or other granulomatous manifestations, certain “red flags” should prompt a thorough immunological evaluation, including functional and genetic testing. These include early onset of symptoms, particularly in childhood, but also atypical multisystem involvement, such as hepatic and bone marrow infiltration, or absent lung involvement; poor response to conventional therapies, such as steroids; a family history of immunodeficiency; history of recurrent infections; and the isolation of pathogens, especially if atypical mycobacteria. Microbiological cultures of biopsy specimens, thus, should be prioritized as a standard diagnostic step before proceeding to advanced diagnostic or therapeutic interventions. These steps have significant prognostic and therapeutic relevance, as empirical immunosuppression may aggravate unrecognized latent infections. Our case, indeed, illustrates how pathogen identification within sarcoid-like lesions can shift the diagnostic and therapeutic approach, with a profound impact on both the clinical course and the patient’s quality of life.

This report also underscores the need of a multidisciplinary approach involving also immunologists, rheumatologists, onco-hematologists, and pathologists to improve diagnostic accuracy, optimize management, and advance understanding of the relationship between IEI and granulomatous diseases in pediatric patients.

## Data Availability

The raw data supporting the conclusions of this article will be made available by the authors, without undue reservation.
