# Supplementary material for: Uncovering hidden immune defects in childhood granulomatous disorders: a case report
Source: Front Immunol. 2025 Jul 18;16:1634661. doi: 10.3389/fimmu.2025.1634661 (PMC12313637; doi:10.3389/fimmu.2025.1634661)
Supplement: Supplementary file 1 [file DataSheet1.pdf]

Uncovering Hidden Immune Defects in Childhood Granulomatous Disorders: A Case Report

Supplementary material

Table 1, Supplement

	First immunological evaluation	Last immunological evaluation (+ 6 months)
**CD19+**	366 (n.v. 200-500/mmc)	558 (n.v. 200-500/mmc)
**CD3+**	1322 (n.v. 800-2500/mmc)	1924 (n.v. 800-2500/mmc)
**CD3+CD4+**	715 (n.v. 400-2100/mmc)	997 (n.v. 400-2100/mmc)
**CD3+CD8+**	532 (n.v. 300-1300/mmc)	797 (n.v. 300-1300/mmc)
**CD3-CD16+CD56+**	252 (n.v. 70-1200/mmc)	341 (n.v. 70-1200/mmc)
**IgG**	1170 (n.v. 600-1310 mg/dL)	924 (n.v. 600-1310 mg/dL)
**IgA**	270 (n.v. 30-220 mg/dL)	193 (n.v. 30-220 mg/dL)
**IgM**	217 (n.v. 50-180 mg/dL)	141 (n.v. 50-180 mg/dL)
**IgE**	2434 (n.v. <100 KU/L)	271 (n.v. <100 KU/L)
**CD19+CD24hiCD38hi**	0%	9.2%
**CD4+CD45RA**	8%	17%
**CD4+CD45RO**	92%	83%
**CD4+CD45RA+C31+**	67%	68%
**CD19+CD27+ IgD-**	4.6%	3.7%
**IFNγR**	99%	
**IGRA**	Negative	
**DHR test**	Negative (Neutrophils Stimulation Index 347)	
**Proliferation assay**	98% (IL2) 99% (PHA)	

Immunological laboratory tests performed in the patient with STAT1 deficiency.

Figure 1, Supplement

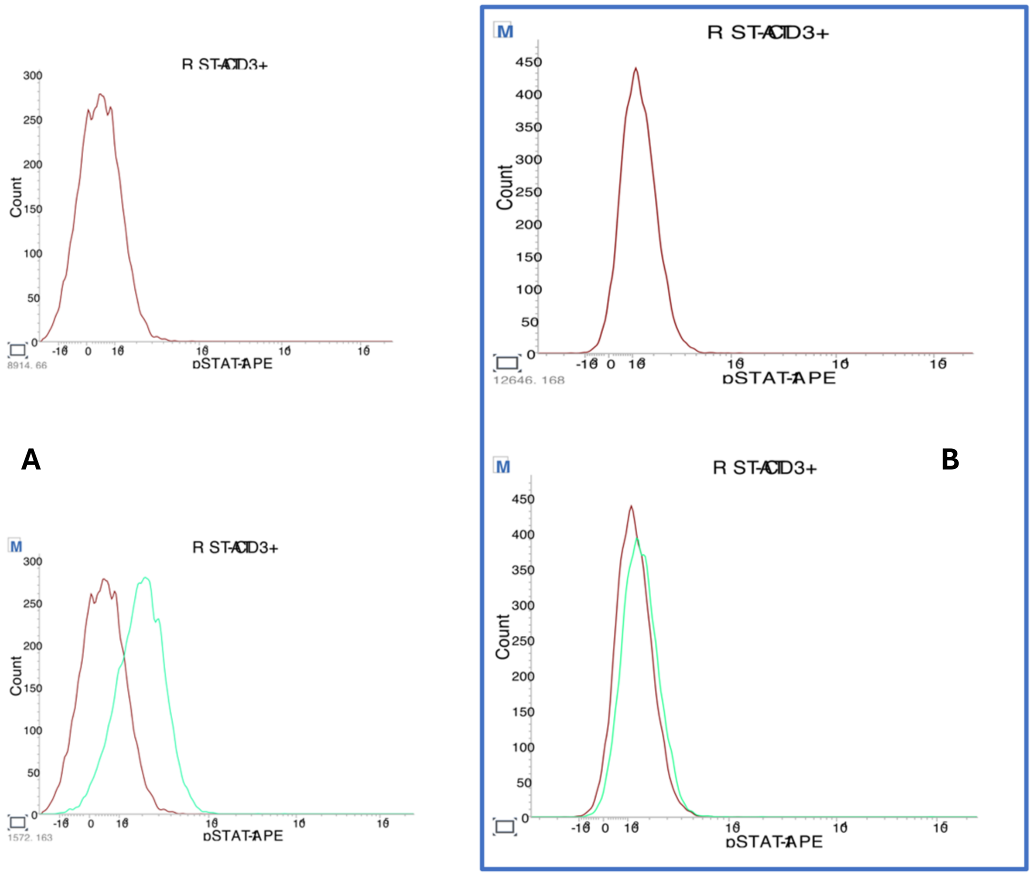

Flow cytometric analysis of STAT1 phosphorylation (pSTAT1) in CD3+ T- in response to high-dose IFNγ. Representative flow plots showing % pSTAT1+ cells in (A) healthy control and (B) Patient with AD STAT1 deficiency. Peripheral blood mononuclear cells (PBMCs) were stimulated with IFNγ (1000 IU/ml) or IFNα (40000 U/mL). for 15 min. Intracellular phosphorylated STAT1 (pSTAT1) was evaluated by gating on CD3+ and CD14+ cells in PBMCs stimulated with IFNα and CD14+ cells in PBMCs stimulated with IFNγ. Figure 3A shows increase in STAT1 phosphorylation after stimulus whereas 3B shows no significant changes.

Methods, Supplement

Patient

A female patient with sarcoidosis-like symptoms was diagnosed with MSMD at Meyer Children's Hospital IRCCS, Florence, Italy. Following written informed consent from her parents, peripheral blood samples were obtained from the patient and her healthy parents. Clinical history, laboratory data, and imaging results were systematically reviewed, and the patient was treated and monitored accordingly with regular follow-up.

Functional assays

PBMCs from patient and healthy control were left unstimulated or stimulated with IFNγ (1000U/mL- Milteny) or IFNα (40000 U/mL, BD Biosciences). Incubation was performed for 15 minutes at 37°C and 5% CO2. The cells were then fixed with Phosflow Fix buffer I at 37°C for 10 minutes and permealized with Phosflow Perm Buffer II according to the BD protocol on ice for 30 minutes. The staining was performed with CD3 Pe Cy7 (BD Biosciences), CD4 APC (BD Biosciences) for lymphocytes and CD14 APC Cy7 for monocytes. The intracellular antibody PE-conjugated anti-phospho*STAT1* (clone pY701 - BD Biosciences) was added together with the other antibodies to detect the phosphorylated *STAT1*. The samples were acquired by flow cytometry (FACS Lyric-BD Biosciences) and the data analysis was performed using Software FACSuite v1.5.

Sanger Sequencing

Universally tagged sequencing primers were designed using a specific software. Genomic DNA was amplified by singleplex PCR. Thermal cycling was performed with 15 cycles [30 seconds at 98°C; 30 seconds at 62°C (−0.5°C each cycle); 60 seconds at 72°C], followed by 15 cycles (30 seconds at 98°C; 30 seconds at 55°C; 60 seconds at 72°C). The raw data obtained from the sequencer (Applied Biosystems™ 3500 Dx Genetic Analyzer) were assembled and analyzed using dedicated software to obtain the nucleotide sequence.

Next Generation Sequencing (NGS)

According to International Union of Immunological Society 2022 classification of Inborn Errors of Immunity11, NGS targeted multigene panel tests covering 74 genes according to those listed in tables of defective innate or intrinsic immunity were used. The panel was designed to

cover the exons, UTRs, and exon-intron junctions with a minimum of 100 intronic bases at the flanking ends. The sequencing assay setup and the subsequent analysis were validated using control samples with known mutations identified by Sanger sequencing. Finally, tertiary analysis was done through the BaseSpace Variant Interpreter software, which enabled the identification of biologically significant variants. The interpretation of these was realized through an integrated knowledge base containing genotype-phenotype associations.
